# Nanoinformatics: Emerging Databases and Available Tools

**DOI:** 10.3390/ijms15057158

**Published:** 2014-04-25

**Authors:** Suresh Panneerselvam, Sangdun Choi

**Affiliations:** Department of Molecular Science and Technology, Ajou University, Suwon 443-749, Korea; E-Mail: sureshcbt@gmail.com

**Keywords:** biomedical informatics, databases, molecular simulations, nanoinformatics, nanomedicine, nanotechnology, QSAR, text mining

## Abstract

Nanotechnology has arisen as a key player in the field of nanomedicine. Although the use of engineered nanoparticles is rapidly increasing, safety assessment is also important for the beneficial use of new nanomaterials. Considering that the experimental assessment of new nanomaterials is costly and laborious, *in silico* approaches hold promise. Several major challenges in nanotechnology indicate a need for nanoinformatics. New database initiatives such as ISA-TAB-Nano, caNanoLab, and Nanomaterial Registry will help in data sharing and developing data standards, and, as the amount of nanomaterials data grows, will provide a way to develop methods and tools specific to the nanolevel. In this review, we describe emerging databases and tools that should aid in the progress of nanotechnology research.

## 1. Introduction

Nanotechnology enables the design and assembly of components with dimensions ranging from 1 to 100 nanometers (nm) [1]. The word nano, which is derived from the Greek word for dwarf, has been part of the scientific nomenclature since 1960 [2]. Macroparticles, which are particles with dimensions greater than 100 nm, have properties that are comparable to those of bulk materials; however, when particles are smaller than 100 nm, their behavior is influenced by atomic, molecular, and ionic interactions. Nanoparticles show novel and changeable properties due to quantum effects [3]. The material properties at the nanoscale level are unusual for two major reasons. First, nanomaterials have a considerably larger surface area than the equivalent mass of a material in a larger form. For instance, if we cut a one centimeter cube into 10^21^ cubes of one nm each, although the overall mass and volume remains the same, the surface area will increase by one million-fold [3]. Second, at the nanoscale level, quantum effects can begin to dominate the behavior of matter, thereby affecting the optical, electrical, and magnetic properties of materials. This behavior is the result of the actions of the electrons in the nanomaterials. Electrons possess two key characteristics [3]: rotation and a capacity to shift among particular energy levels. In iron oxide magnetic nanoparticles (≤20 nm in diameter), electrons rotate in the same direction, whereas in iron oxide macroparticles (>20 nm in diameter), electrons rotate in opposite directions. When electron spins are aligned in the same direction, the fields are additive, but as soon as the electrons begin to rotate in different directions, the fields eliminate each other. The total magnetic field strength of a nanomaterial is the sum of the magnetic fields of individual electrons. Consequently, nanoparticles comprise greater localized magnetic fields than larger particles [3].

**Figure 1.**
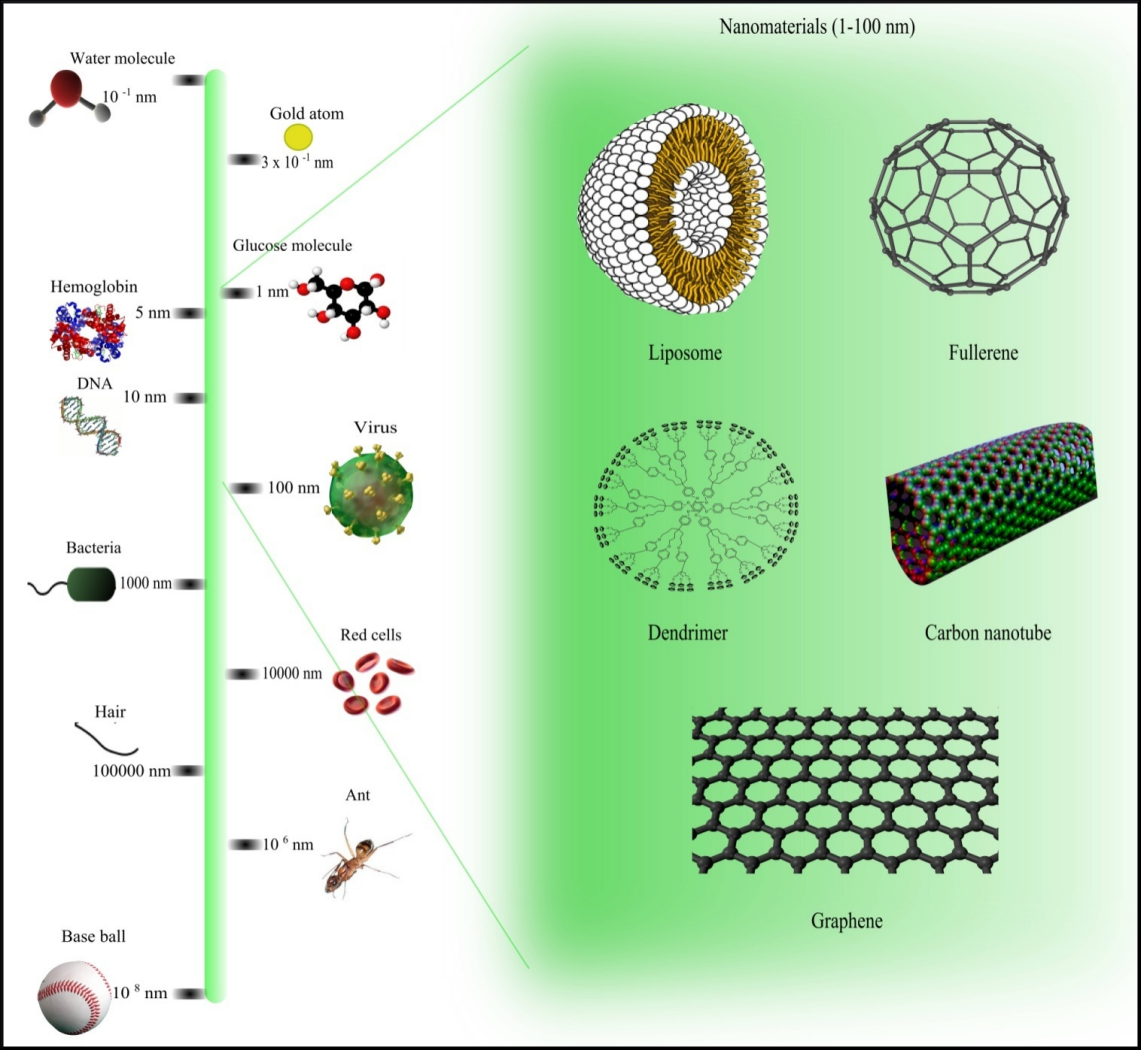
Comparison of the sizes of nanomaterials with those of other common materials. Figures were drawn using Inkscape [20] and GNU Image Manipulation Program (GIMP) [21]. All the images in this figure were obtained from Wikimedia Commons.

In general, nanoparticles can be classified into three categories: organic, inorganic, and hybrid particles. Simple monomeric building blocks such as polymer-based nanoparticles are organic particles, whereas inorganic nanomaterials include carbon nanotubes, fullerene particles, and metallic nanoparticles (gold and silver nanoparticles). Hybrid nanoparticles comprise engineered nanoparticles such as DNA-carbon nanotube arrays [4]. Because the sizes of nanomaterials are comparable to the sizes of biomolecules such as proteins, they can be designed for biomedical applications such as devices for diagnosis, drug delivery, and therapy [5]. The sizes and shapes of some nanomaterials as compared to those of common materials are shown in Figure 1 [3,6]. Nanoparticles are found in many consumer products such as sheets and clothing (silver nanoparticles), cosmetics and sunscreens (titanium dioxide), bicycles (carbon nanoparticles), and even beer bottles (clay nanoparticles) [7]. Doxil and Abraxane, which are currently available on the market, are Food and Drug Administration-approved nanoformulations for cancer treatment [8]. Although the unique properties of nanomaterials hold promise for many applications, they also raise safety concerns. Nanoparticles can pose risks for human health because of their ability to penetrate into the human body, flow in the bloodstream, and pass through cells, which may cause undesirable side effects [9]. Therefore, knowledge of the biological and toxicological effects of nanomaterials is essential to determine whether the products are safe. However, assessing the safety of nanomaterials through experiments is costly and time consuming; hence, computational methods offer a profitable alternative [10]. Computational approaches can not only serve to build binding models of protein-nanoparticle complexes, but can also be used to simulate the binding processes [11].

Nanoinformatics is described as “the science and practice of determining which information is relevant to the nanoscale science and engineering community, and then developing and implementing effective mechanisms for collecting, validating, storing, sharing, analyzing, modeling and applying that information” [12]. The major setback of the discipline of nanoinformatics is that it is still at an early stage of development and the data are scattered in the form of publications [13–15]. Recently, some initiatives have emerged in the field of nanoinformatics with respect to nanoparticle data management [16–19]. The goal of this review is to describe the emerging databases and tools available for the nanotechnology community. We have categorized the nanoinformatics tools in this review based on relevant examples provided by Maojo *et al.* as well as on the availability of data [13].

## 2. Growth of Bioinformatics and Applications for Nanoinformatics

In the 1980s, it became mandatory to deposit all published DNA sequences in a central repository. At the same time, a standard was adopted by journals to make gene and protein sequences freely accessible to all and to compile all data in the form of openly available databases. The open accessibility of DNA sequences in databases such as GenBank [22] and the availability of protein structures in databases such as the Protein Data Bank [23] have motivated many researchers to develop powerful methods, tools, and resources for large-scale data analysis [24]. Simultaneously, growth in computational speed and memory storage capacity has led to a new era in the analysis of biological data. Bioinformatics has emerged as a powerful discipline and almost 1000 databases are currently publicly available [25] along with a large number of bioinformatics tools [26]. The growth and development of bioinformatics can provide valuable lessons for applying the same practice to benefit nanoinformatics development [27].

## 3. Databases

An overview of nanomaterial-related databases is provided in Table 1. The emergence of recent databases such as Investigation Study Assay (ISA) tab-delimited (TAB) format (ISA-TAB-Nano) [19], Cancer Nanotechnology Laboratory (caNanoLab) [17], and Nanomaterial Registry seems encouraging and is discussed below.

### 3.1. ISA-TAB-Nano

Motivated by an absence of standardization in representing and distributing nanomaterial data, the ISA-TAB-Nano was established for the public development of data sharing [19]. The ISA-TAB-Nano file format offers a common framework to record and integrate nanomaterial descriptions and uses the ISA-TAB open-source framework file format. ISA-TAB-Nano defines four file formats for the distribution of the data: (1) the Investigation file; (2) the Study file; (3) the Assay file; and (4) the Material file. The Investigation file provides reference data about each investigation, study, assay, and protocol. The Study file contains the names and attributes of procedures applied for preparing samples for examination (sources and characteristics of biospecimens). The Assay file contains the values of measured endpoint variables and references to external files for each analyzed sample. The Material file describes the material sample and its structural and chemical components. File format templates are available [36], which also provide information on using ontology terms to support standardized descriptions and assist searches for and incorporation of information. Ontology is a formal way of representing knowledge abstracted from the growing body of biological science in a coded form, which can be translated into a programming language [43,44]. A benefit of ontologies is to facilitate the description logic that can be utilized together for querying a dataset. Moreover, the data sets are analyzed which are not usually accessible to searching and comparing. Ontologies are used in databases in various ways. For example, ontologies are characterized in a computer-readable form that can be interpreted by computers as well as domain experts. Thomas *et al.* have provided a review of ontology terms used for standardized descriptions [45]. A list of ontology-related resources for nanotechnology is shown in Table 2.

### 3.2. caNanoLab

**Figure 2.**
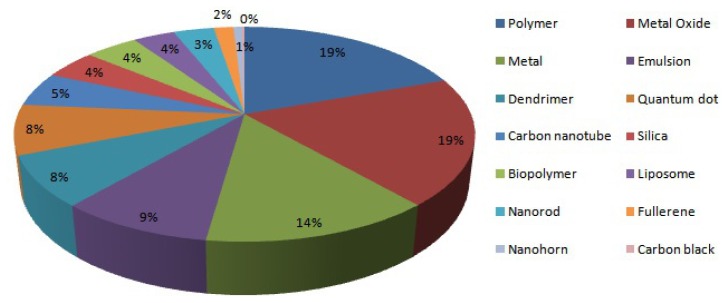
Summary of data available from caNanoLab.

caNanoLab is a data repository that researchers can use for the submission and retrieval of information on nanoparticles, including their composition, function (e.g., therapeutic, targeting, diagnostic imaging), physical (e.g., size, molecular weight), and *in vitro* experimental characterizations (e.g., cytotoxicity, immunotoxicity) [17]. It also encourages data sharing and analysis across the cancer community to accelerate and authenticate the use of nanoparticles in biomedicine. To facilitate data submission, web-based forms are offered so that submitters can restrict the visibility of their records to be private, to be distributed to particular collaboration groups, or to be public. In addition, caNanoLab can be utilized for discovery purposes by using it to search publicly accessible physical and *in vitro* characterizations while providing access to the associated publications. Moreover, the query results are downloadable in spreadsheet-based formats. The caNanoLab project team is also currently engaged with ISA-TAB-Nano to facilitate the submission and exchange of data across the breadth of nanotechnology information. Future features of caNanoLab may include support for the validation, import, and export of ISA-TAB-Nano files by means of customized ISA-TAB tools. A summary of data available from caNanoLab is presented in Figure 2.

### 3.3. Nanomaterial Registry

**Figure 3.**
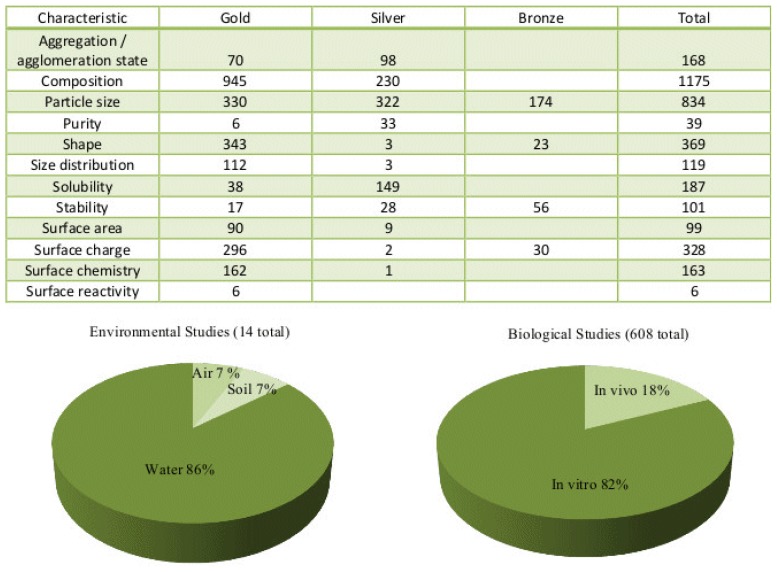
Data available from the Nanomaterial Registry.

For data to be useful to a wide community of researchers, the data must be easily available in a usable form. However, providing usable data is a task that becomes increasingly difficult as the quantity of information grows. Curation is particularly important for nanomaterials because the experimental conditions and process of sample preparation can affect the actual data measurement. Data curation is best achieved through increasing proficiency in assessing a specific feature of a specific dataset. To increase the efficiency of the information curation method, the Nanomaterial Registry developed a method to assist the combinatorial analysis of a variety of datasets [16]. The Nanomaterial Registry is a web-based database that offers curated data for characterized nanomaterials. The web-based curation tool has a dual functionality that maintains (a) manual data entry and (b) programmatic data upload. The curation tool resides in a protected environment that contains user accounts, roles, and permissions. User roles are supported by an administration tool that offers access, controls, and permissions for curators. As the curation technique improves and the dataset grows, these resources will become increasingly available to researchers and information analysts. Data available from the Nanomaterial Registry are shown in Figure 3.

## 4. Text Mining

Text mining is a computerized process for utilizing the enormous amount of knowledge existing in the literature [56,57]. Three important approaches to text mining are currently prevalent in the field of biology: (1) co-occurrence-based methods; (2) rule-based or knowledge-based approaches; and (3) statistical or machine learning-based approaches. Co-occurrence-based methods scan for specific terms or ideas that occur in the same sentence but are sometimes abstract, and then hypothesize an association between them. Rule-based systems make use of common knowledge about how language is structured, specific knowledge about how biologically significant information is stated in the literature, and the relationships that these two types of knowledge can have with one another. In contrast, statistical or machine learning-based systems function by building classifiers that may operate at any level. However, free full-text access for a large proportion of scientific journals is still unavailable. In several disciplines, such as chemistry, it is still hard to find enough abstracts for large-scale analysis [56,57]. Although the application of text mining to nanotechnology is at an early stage, some recent studies have been published [58–60]. The few available text-mining tools are as follows: (1) the Google Scholar search engine [61]; (2) the GoPubMed engine [62]; (3) Textpresso [63]; and (4) BioRAT [64]. When literature is available for analysis, it is possible to predict interactions via text mining, for example, using iHOP [65], which is an online service through which the names of genes and proteins in sentences from abstracts in PubMed can be hyperlinked and assembled into a conceptual network. Likewise, it might be possible to use text mining to predict nanoparticle-protein interactions for nanotechnology.

## 5. Molecular Modeling

The structures of nucleic acids and many proteins have been determined by crystallography, nuclear magnetic resonance, electron microscopy, and many other techniques. Structural biology provides information on the static structures of biomolecules. However, in reality, biomolecules are highly dynamic, and their motion is important to their function. Different experimental techniques are available to help study the dynamics of biomolecules. Computational power continues to increase, and the development of new theoretical methods offers hope of solving scientific problems at the molecular level. All the theoretical methods and computational techniques that are used to model the behavior of molecules are defined as molecular modeling. Macromolecules can be studied only with molecular mechanics since other quantum methods based on the Schrodinger equations such as *ab initio*, semi-empirical, and density functional theory (DFT) methods require extensive computational time. Molecular mechanics uses classical physics and relies on force field. *Ab initio* and semi-empirical methods are based on approximate solutions of the Schrodinger equation. In addition, semi-empirical methods need additional empirical parameters (*i.e.*, using parameterization) for the calculations. DFT methods are based on electron density. Novel molecules are best studied with *ab initio* or possibly DFT calculations, since the parameterization intrinsic to molecular mechanics or semi-empirical methods makes them unreliable for molecules that are different from those used in the parameterization. The energies of molecules such as proteins or DNA can be calculated using molecular mechanics; small molecule energies can be calculated using *ab initio* and semi-empirical methods [66,67]. There is an extensive body of three-dimensional structures of molecules essential to molecular modeling that can be visualized using software freely available for users for this purpose. A list of software for visualizing macromolecules is presented in Table 3.

### 5.1. Docking

Docking is an important computational tool in the drug discovery process and is used to specifically predict protein-ligand interactions. The two basic features of docking software are docking accuracy and scoring reliability [84]. Docking accuracy indicates how similar the prediction of ligand binding is to the ligand conformation that is determined experimentally, whereas scoring reliability ranks ligands based on their affinities. Docking accuracy and scoring reliability are used to assess the searching algorithm and the scoring functions, respectively, of docking software. The numerous searching algorithms used in docking software vary with respect to randomness, speed, and the area covered. Most of the searching algorithms perform well when tested against the known structure. In contrast, scoring functions are rarely successful, and a number of issues needed to be addressed to improve the docking features. Many types of docking software are currently available; AutoDock is a software that is highly accessed and freely available [85]. At present, as protein-nanoparticle complexes are difficult to study using experimental approaches, computational tools show promise. Structural models for carbon nanomaterials such as carbon nanotubes and fullerenes are available, and thus many protein-nanoparticle interactions can be studied computationally. Fullerenols, which are derivatives of fullerenes, are currently in trial for diagnostic and therapeutic uses, although insufficient information is available about the structural interactions and toxicity of fullerenols in biosystems. Yang *et al.* computationally studied the interactions in the fullerenol-lysozyme complex and compared the computational results with experimental results [86]. The structure of fullerenol was constructed using Chem3D Ultra and subjected to blind docking in the active site of chicken lysozyme. The fullerenol bound close to Tryptophan 62, and a π–π stacking interaction was observed. The predicted computational binding result was consistent with all known experimental results. As a result, new proteins that can interact with fullerenol can be identified for future applications as drug targets [87]. The addition of molecular dynamic (MD) simulations to docking studies can also assist in studying interactions. A list of well-known docking software is provided in Table 4.

### 5.2. Quantitative Structure-Activity Relationships (QSAR)

QSAR is based on the idea that similar structural properties generate similar biological effects [94]. It is used to establish associations between the structural and electronic properties of potential drug candidates along with their binding affinities for common macromolecular targets that are widely used in drug discovery. QSAR studies were originally based on a single physicochemical property such as the solubility or the pKa (acid dissociation constant) value to elucidate the biological effect of a molecule (one-dimensional QSAR). Two-dimensional QSAR further examines the connectivity of a compound by considering the physicochemical properties of single atoms and functional groups and their roles in biological activity. Current models contain three-dimensional structural descriptors such as the length or width of a substituent. With a suitable force field, it is theoretically possible to determine the feasible binding mode of any given existing molecule to a target protein. Most three-dimensional QSAR models depend on the superposition of ligands, the identification of which is impossible without sufficient structural information for the target protein. The results of QSAR can be used to understand the interactions occurring between functional groups in the molecules with those of their target. QSAR methods are fast and can deal with real complex biological systems. QSAR models provide an alternative to animal testing and offer the advantages of reduced time for experimentation and minimized costs [95]. A few QSAR studies related to nanoparticles have been published to date [96–100]. QSAR can also predict the toxicity of nanoparticles. In addition, it can support targeting and filling gaps in knowledge for known nanoparticles [101]. QSAR tools are presented in Table 5. Recent reviews provide clear future directions for the field [102,103].

#### High-Throughput Screening Data Analysis Tools (HDAT)

HDAT is a set of web-based high-throughput screening (HTS) data analysis tools [110,111]. The use of HTS in toxicity studies of engineered nanomaterials requires tools for quick and consistent processing and analyses of large HTS datasets. To facilitate this, a web-based platform was developed that presents statistical methods appropriate for toxicity data on engineered nanomaterials. It also offers different plate normalization methods, different HTS summarization statistics, self-organizing map (SOM)-based clustering analysis, and visualization of raw and processed information using heat maps and SOMs. HDAT has been successfully applied in the analysis of a number of HTS studies on the toxicity of engineered nanomaterials, thus enabling analysis of toxicity mechanisms and the development of data-driven structure-activity relationships for nanomaterial toxicity. HDAT offers online instructions and video demonstrations of basic operations such as data formatting and data uploading.

### 5.3. MD Simulations

The most broadly used computational technique is MD simulation, which elucidates the structure-function relationships in biomolecules. In MD simulation, atoms and molecules are allowed to interact over time at a given temperature and are assumed to follow the laws of classical mechanics. MD simulation offers a detailed description of atomic motion, and the forces acting on the atoms are computed using a model known as a force field [158]. Such a simulation may serve as a “computational microscope” [159] that reveals interactions that may be difficult to observe experimentally. Dror *et al.* explained MD simulation as follows: “Static structural information might be likened to a photograph of a football game; to understand more readily how the game is played, we want a video recording” [159]. Computer simulation of biomolecule-nanomaterial interactions is gaining popularity as a complement to experimental techniques. When entering human bodies, nanoparticles interact with biomolecules and begin a series of nanoparticle/biological interactions that rely upon on forces as well as dynamic biophysicochemical interactions [11]. Many interactions such as hydrogen bonds, Van der Waals (VDW) forces, and electrostatic, hydrophobic, and π–π stacking interactions contribute to these protein-nanoparticle complexes [11]. The VDW attraction increases with increase in the proximity of the atoms at the interface, within a distance close to the total of the VDW radii of the two atoms. The VDW force acts at a short range and reduces significantly as the contacting atoms depart (Lennard-Jones potential or 6–12 potential). Hydrogen bonding is another important player in intermolecular interactions. Although the hydrogen bond is much stronger than the VDW interaction, the number of hydrogen bonds connecting proteins and nanoparticle is much smaller than that of VDW interactions. Hydrophobic interactions are an entropic effect originating from the repulsion of ordered water molecules from a nonpolar surface. The π–π stacking is an attraction involving aromatic rings. However, as only a few amino acids contain aromatic rings and the rings are often buried within the hydrophobic core of the proteins, only specific interactions are possible. A recently published review of molecular modeling in structural nanotoxicology discusses methods for predicting interactions between nanomaterials and biomolecules [160]. The review also discusses how nanoparticles of different sizes, shapes, structures, and chemical properties are able to influence the organization and function of nanomachinery in cells. In summary, computer modeling studies have become a dominant tool in risk assessment studies. The molecular modeling tools currently available are shown in Table 6.

## 6. Imaging

Medical imaging technologies are used for diagnosis as well as for many other biomedical applications. The use of nanomaterials in imaging applications is growing rapidly. Molecular imaging can assist in early diagnosis and also provides information on pathological processes. Compared with currently used fluorescent proteins and small-molecule dyes, nanotechnology imaging probes can offer signals that are several-fold brighter and more stable. Nanoparticles, predominantly those made of precious metals, enhance signals in imaging approaches. For example, semiconductor quantum dots are tiny light-emitting particles that are used as fluorescent probes for molecular imaging and medical diagnostics. However, there are still many disadvantages associated with imaging technologies, such as tissue specificity and systemic toxicity. Progress in nanotechnology may overcome these challenges and offer more sensitive and specific information [177]. *In vivo* and *in vitro* imaging applications of nanomaterials have been reviewed [3,178]. The new informatics methods link tissue banks with histology data in order to offer enhanced image annotation down to the nano level [13]. Due to the increase in the use of image analysis tools and methods in medical applications, well-supported tools and databases that can be used for nanotechnology have become increasingly available; these are shown in Table 7 [179]. Image databases and tools used in fluorescence microscopy can also be used to enhance image annotation with novel methods as the amount of experimental data at the nanolevel grows.

## 7. Computational Design of Biomolecular Nanostructures

DNA and protein self-assemblies are used to build nanostructures because of the programmability property of DNA and protein and the surplus of chemical techniques available for their manipulation. For example, DNA is a valuable self-assembly material because, with appropriate design of its base sequences, assembly is enabled through specific interactions among complementary base pairs [180]. Many self-assembly molecules that can be used as vehicles for drug delivery are built using DNA [181]. Some of the software currently available for designing three-dimensional structures of carbon nanotubes and for DNA origami design is listed in Table 8.

## 8. Specific Challenges and Opportunities in Nanoinformatics

The databases and tools presented here highlight the increasing resources that are available to users. Their improvement is accompanied by the continuous expansion in the number of databases (Table 1) and various computer programs (Tables 6 and 7). The new database initiatives such as ISA-TAB-Nano, caNanoLab, and Nanomaterial Registry will facilitate data sharing, data standards, and, depending on the growth of nanomaterials data, the development of methods and tools specific to the nanolevel. Moreover, the growth of these fields essentially requires that the ISA-TAB-Nano standard be adopted by journals and other organizations to ensure consistent representation of nanotechnological data [19]. It should be noted that open accessibility and the liberty to use published DNA sequences in databases such as GenBank has encouraged scientists to build powerful methods, tools, and resources that have substantially enriched the field of bioinformatics [24]. It should also be noted that nanomaterial data are much more complex than sequence information or molecular data. An additional challenge is the lack of availability of scientific literature from subscription-based journals in nanotechnology, particularly in chemistry, for which even abstracts are inaccessible for large-scale analyses such as text mining; this is a big drawback for text mining and nanoinformatics analysis [193]. With respect to QSAR, a recent review provided a summary of the advances and a clear roadmap for future research and also identified major gaps in the field [102]. Molecular modeling and imaging in nanotoxicology appear promising, as many software tools mentioned in this review are free under the General Public License, so that that there is freedom to use, study, share, and modify the software. Although the modeling behavior of nanomaterials presents different challenges compared to those for drugs, it is nonetheless possible to adapt new methods and develop specific tools from existing tools. There are also anticipated beneficial side effects of nanoinformatics development, such as the possible minimization of animal studies. Recent initiatives and efforts emerging from the nanotechnology community are encouraging.

## 9. Conclusions

The databases and tools presented herein will be helpful to the nanotechnology community. We highlight the following recommendations that we hope will assist this community: (i) Consensus standards and ontology terms should be used in nanotechnology-related research; (ii) Journals should adopt formats to ensure uniform representation of information; (iii) Authors should submit their data to the databases; (iv) Similar to the current model of molecular biology databases, free and unrestricted access to nanomaterial data would enable nanoinformatics researchers to create tools that will benefit the broad nanotechnology community; (v) Open access and freely available nanotechnology literature will also benefit the nanotechnology community.

## Figures and Tables

**Table 1.** Summary of nanotechnology-related databases.

No.	Name	Description	Url
1	Nanomaterial Biological Interactions Knowledgebase	The knowledgebase serves as a repository for annotated data on nanomaterial characterization (purity, size, shape, charge, composition, functionalization, and agglomeration state), synthesis methods, and nanomaterial-biological interactions.	[28]
2	InterNano	InterNano supports the information needs of the nanomanufacturing community by bringing together resources related to advances in applications, devices, metrology, and materials that will facilitate the commercial development and/or marketable application of nanotechnology.	[29]
3	Nano-EHS Database Analysis Tool	This web tool provides a quick and thorough synopsis of the Environment, Health and Safety Database.	[30]
4	Nanoparticle Information Library	The goal of the NIL is to help occupational health professionals, industrial users, worker groups, and researchers organize and share information on nanomaterials, including their health and safety-associated properties.	[31]
5	Nanomaterials Registry	The Registry was established to provide researchers with a convenient data management and sharing plan.	[32]
6	National Toxicology Program Database	The goal of this database is to develop and apply modern toxicology and molecular biology tools to identify substances in the environment that may affect human health.	[33]
7	Nano-HUB database	Nano-HUB offers a searchable online database of nanoBIO tools.	[34]
8	National Center for Biomedical Ontology Bioportal	A major focus involves the use of biomedical ontologies to aid in the management and analysis of data derived from complex experiments.	[35]
9	ISA-TAB-Nano	The ISA-TAB-Nano standard specifies the format for representing and sharing information about nanomaterials, small molecules, and biological specimens along with their assay characterization data (including metadata and summary data) using spreadsheet or TAB-delimited files.	[36]
10	caNanoLab	caNanoLab is a data-sharing portal designed to facilitate information sharing in the biomedical nanotechnology research community to expedite and validate the use of nanotechnology in biomedicine.	[37]
11	Toxicology Data Network	Includes databases on toxicology, hazardous chemicals, environmental health, and toxic releases.	[38]
12	Nanotechnology Characterization Laboratory	The Nanotechnology Characterization Laboratory performs and standardizes the preclinical characterization of nanomaterials intended for cancer therapeutics and diagnostics developed by researchers from academia, government, and industry.	[39]
13	Collaboratory for Structural Nanobiology	A nanoinformatics service dedicated to the collection, curation, and correlation of structural, physicochemical, and biological and biomedical data.	[40]
14	Toxicology Literature Online	Provides references from the toxicology literature.	[41]
15	OECD	Database on research into the safety of manufactured nanomaterials.	[42]

**Table 2.** List of nanoparticle-related ontology resources.

No.	Name	Description	Url
1	NanoParticle Ontology	The NPO was developed to provide knowledge regarding the description, preparation, and characterization of nanomaterials in cancer nanotechnology research.	[46]
2	BioPortal	BioPortal provides access to commonly used biomedical ontologies and tools for their analysis.	[47]
3	The Open Biological and Biomedical Ontologies	A collaborative experiment involving developers of science-based ontologies who are establishing a set of principles for ontology development with the goal of creating a suite of orthogonal interoperable reference ontologies in the biomedical domain.	[48]
4	Cancer Open Biomedical Resource	A tool for indexing cancer nanotechnology informatics knowledge with open biomedical resources.	[49]
5	Chemical Entities of Biological Interest	A database and ontology of chemical entities of biological interest.	[50]
6	Gene Ontology	The Gene Ontology project is a major bioinformatics initiative with the aim of standardizing the representation of gene and gene product attributes across species and databases.	[51]
7	Foundational Model of Anatomy ontology	The Foundational Model of Anatomy Ontology is an evolving computer-based knowledge source for biomedical informatics.	[52]
8	Zebrafish Anatomy and Development ontology	A structured, controlled vocabulary of the anatomy and development of the zebrafish.	[53]
9	Ontology Lookup Service	Provides a web service interface to query multiple ontologies from a single location with a unified output format.	[54]
10	Phenotype Quality ontology	Phenotype Quality provides ontology of phenotypic qualities intended for use in a number of applications, primarily defining composite phenotypes and phenotype annotation.	[55]

**Table 3.** List of visualization tools.

No.	Name	Operating System	License	Url
1	PyMOL	Windows/GNU Linux	Free and commercial	[68]
2	RasMol	Windows/GNU Linux	GNU General Public License	[69]
3	Raster3D	GNU Linux/Unix/Mac OS X	Artistic License	[70]
4	UCSF Chimera	Windows/GNU Linux/Mac OS X	Free for non-commercial use	[71]
5	VMD	Windows/GNU Linux/Mac OS X/Solaris	VMD License/Free to use	[72]
6	Cn3D	Windows/Mac OS X	Free to use	[73]
7	BALLView	Windows/GNU Linux/Unix/Mac OS X	GNU Lesser General Public License	[74]
8	Jmol	Windows/GNU Linux/Mac OS X	GNU Lesser General Public License	[75]
9	ICM-Browser	Windows, Mac, and GNU Linux	Commercial software	[76]
10	Chemkit	GNU Linux	BSD licenses (free to use)	[77]
11	OpenStructure	GNU Linux/Mac OS X	GNU Lesser General Public License	[78]
12	VEGA ZZ	Windows/GNU Linux/Mac OS X/Silicon Graphics/Amiga OS	Copyright (free for non-profit academic)	[79]
13	Molekel	Windows/GNU Linux/Mac OS X	GNU General Public License	[80]
14	Avogadro	Windows/GNU Linux/Mac OS X	GNU General Public License	[81]
15	Xeo	Windows/GNU Linux/Mac OS X	GNU General Public License	[82]
16	ChemSketch	GNU Linux/Mac OS X	Commercial software (free for academic and personal use)	[83]

**Table 4.** List of tools available for docking.

No.	Name	Operating System	License	Url
1	Autodock 4	Windows/GNU Linux/Mac OS X	GNU General Public License	[88]
2	AutoDockVina	Windows/GNU Linux/Mac OS X	Apache license	[89]
3	Dock	Windows/GNU Linux/Mac OS X	Academic software license	[90]
4	GOLD	Windows/GNU Linux	Commercial software	[91]
5	FlexX	Windows/GNU Linux	Commercial software	[92]
6	Surflex-dock	Windows/GNU Linux	Commercial software	[93]

**Table 5.** List of tools available for QSAR.

No.	Name	Operating System	Url
1	ChemProp	Windows	[104]
2	OECD ToolBox	Windows/GNU Linux/Mac OS X	[105]
3	RmSquare	Web based application	[106]
4	T.E.S.T	Windows/GNU Linux/Mac OS X	[107]
5	Virtual Computational Chemistry Laboratory	Few online servers and commercial software	[108]
6	CORAL	Windows	[109]

**Table 6.** List of tools available for molecular modeling.

No.	Name	Operating System	License	Url
**Molecular Dynamic Simulations**				

1	Amber	Unix-like systems	Academic/Non-profit/Government: $400	[112]
2	NAMD	Windows/GNU Linux/Mac OS X/Solaris	Free for academics	[113]
3	Gromacs	Solaris, Linux, OS X, Windows by Cygwin, any other Unix variety	GNU Lesser General Public License for version	[114]
4	CHARMM	Unix-like	Academic research groups for a $600 licensing fee	[115]
5	Desmond	Linux/Windows	Academic and Commercial	[116]
6	LAMMPS	Windows/GNU Linux/Mac OS X	GNU General Public License	[117]
7	TINKER	Windows/GNU Linux/Mac OS X	Available free of charge	[118]
8	MDynaMix	-	GNU Lesser General Public License	[119]
9	CP2K	GNU Linux	GNU Lesser General Public License	[120]

**DFT/*Ab initio*/Monte Carlo/Semi-empirical calculations**				

10	ABINIT	Windows/GNU Linux/Mac OS X	GNU General Public License	[121]
11	ACES	GNU Linux/SGI	GNU General Public License	[122]
12	BigDFT	Cross-platform	GNU General Public License	[123]
13	CASINO	Linux related operating system/Windows by Cygwin	Charge-free license agreements for academics	[124]
14	CASTEP	-	Commercial software	[125]
15	COLUMBUS	GNU Linux	Obtained free of charge	[126]
16	CFOUR	GNU Linux	Charge-free license agreements for academics	[127]
17	CONQUEST	GNU Linux	GNU General Public License	[128]
18	CPMD	GNU Linux	CPMD License (free for non-profit)	[129]
19	CRYSTAL	Windows/GNU Linux/Mac OS X	Commercial software	[130]
20	DACAPO	GNU Linux	CAMP Open Software project	[131]
21	DALTON	GNU Linux	Charge-free license agreements for academics	[132]
22	DFTB+	GNU Linux	Free for academic, educational, and non-profit research	[133]
23	DIRAC	Windows/GNU Linux/Mac OS X	Charge-free license agreements	[134]
24	Elk	GNU Linux	GNU General Public License	[135]
25	ErgoSCF	GNU Linux	GNU General Public License	[136]
26	ERKALE	Windows/GNU Linux	GNU General Public License	[137]
27	FLEUR	GNU Linux	Academic	[138]
28	FreeON	GNU Linux	GNU General Public License	[139]
29	GAMESS	Windows/GNU Linux/Mac OS X	Charge free license agreements	[140]
30	GPAW	GNU Linux/Mac OS X	-	[141]
31	Gaussian	Windows/GNU Linux/Mac OS X	Commercial software	[142]
32	JDFTx	GNU Linux	GNU General Public License	[143]
33	MADNESS	Windows/GNU Linux/Mac OS X	GNU General Public License	[144]
34	MOPAC	Windows/GNU Linux/Mac OS X	Free and Commercial	[145]
35	MPQ	GNU Linux	GNU Lesser General Public License	[146]
36	NWChem	Linux, FreeBSD, Unix and like operating systems, Microsoft Windows, Mac OS X	Educational Community License	[147]
37	Octopus	GNU Linux	GNU General Public License	[148]
38	ONETEP	GNU Linux	Commercial software	[149]
39	OpenAtom	GNU Linux/Mac OS X	Free for academic	[150]
40	OpenMX	GNU Linux	GNU General Public License	[151]
41	ORCA	GNU Linux	Free for academic	[152]
42	PSI	GNU Linux/Windows, Mac OS X	GNU General Public License	[153]
43	PyQuante	-	BSD licenses (free for academic)	[154]
44	Quantum ESPRESSO	GNU Linux	Open source distribution	[155]
45	VASP	GNU Linux	Commercial software	[156]
46	Yambo Code	GNU Linux	GNU General Public License	[157]

**Table 7.** Summary of image-related databases and tools. All tools listed are freely available for use.

No.	Name	Operating System	License	Url
1	Micro-manager	Windows and Mac	Copyright (distributed free of charge)	[161]
2	OMERO	Windows, Mac and Linux	GNU General Public License and few commercial licenses	[162]
3	Bisque	Windows, Mac and Linux	Free and open source	[163]
4	Bio-Formats	GNU Linux	GNU General Public License	[164]
5	ImageJ	Windows, Mac and GNU Linux (any java based)	Public domain	[165]
6	Fiji	Windows, Mac OS X, GNU Linux and Unix	GNU General Public License (the plugin interface is excluded from the license; some plugins have different licenses)	[166]
7	FARSIGHT TOOLKIT	Windows, Mac OS X and GNU Linux	Apache License, Version 2.0	[167]
8	ICY	Windows, Mac OS X and GNU Linux	GNU General Public License	[168]
9	CellProfiler	Windows, Mac OS X, and GNU Linux	GNU General Public License and some portions are also BSD-licensed	[169]
10	Micro-Pilot	Windows	Free for academic users	[170]
12	Cell ID	Windows and GNU Linux	GNU Lesser General Public License	[171]
13	bioView3D	Windows, Mac OS X, and GNU Linux	Open source (free for non-commercial)	[172]
14	V3D	Windows, Mac and GNU Linux	Free for non-profit research	[173]
15	IMOD	Windows, Mac and GNU Linux	Free to use	[174]
16	iCluster	Windows, Mac OS X, and GNU Linux	GNU General Public License	[175]
17	Image Surfer	Windows and Mac	Free to use	[176]

**Table 8.** Programs for DNA origami, nanomaterial design, and carbon nanotube design. All the tools listed are freely available for use.

No.	Name	Operating System	Url
1	Tiamat	Windows	[182]
2	caDNAno	Web-Accessible	[183]
3	SARSE	GNU Linux/Mac	[184]
4	cadnano	Windows, OSX and GNU Linux	[185]
5	Uniquimer3D	Windows	[186]
6	CoNTub	Windows, Mac OS X, GNU Linux and Unix	[187]
7	Fullerene	GNU Linux/Unix and Mac	[188]
8	CS Catalyst	Windows	[189]
9	Ninithi	Windows	[190]
10	TubeGen	Web-Accessible	[191]
11	Wrapping	Windows	[192]
